# Regulation of protein and oxidative energy metabolism are down-regulated in the skeletal muscles of Asiatic black bears during hibernation

**DOI:** 10.1038/s41598-022-24251-0

**Published:** 2022-11-16

**Authors:** Mitsunori Miyazaki, Michito Shimozuru, Yu Kitaoka, Kenya Takahashi, Toshio Tsubota

**Affiliations:** 1 Department of Integrative Physiology, Graduate School of Biomedical and Health Sciences, Hiroshima University, 1-2-3 Kasumi, Minami-Ku, Hiroshima, 734-8553 Japan; 2 Department of Physical Therapy, School of Rehabilitation Sciences, Health Sciences University of Hokkaido, Hokkaido, Japan; 3 Laboratory of Wildlife Biology and Medicine, Graduate School of Veterinary Medicine, Hokkaido University, Hokkaido, Japan; 4 Department of Human Sciences, Kanagawa University, Kanagawa, Japan; 5 Department of Sports Sciences, The University of Tokyo, Tokyo, Japan

**Keywords:** Physiology, Animal physiology

## Abstract

Hibernating animals exhibit an unexplained physiological characteristic of skeletal muscles being atrophy resistance, in which case muscle mass and strength remain almost unchanged both before and after hibernation. In this study, we examined the alterations in the regulatory systems of protein and energy metabolism in the skeletal muscles of Asiatic black bears during hibernation. Skeletal muscle samples (vastus lateralis muscle) were collected from identical individuals (n = 8) during the active (July) and hibernating (February) periods, while histochemical and biochemical analyses were performed. We observed no significant alterations in body weight, muscle fiber size, and fiber type composition during the active and hibernating periods, indicating that the skeletal muscles of bears are very well preserved during hibernation. In hibernating bear skeletal muscles, both regulatory pathways of muscle protein synthesis (Akt/mechanistic target of rapamycin and mitogen-activated protein kinase systems) and proteolysis (ubiquitin–proteasome and autophagy systems) were down-regulated. Gene expression levels of factors regulating oxidative metabolism were also decreased in hibernating bear skeletal muscles. This is likely an adaptive strategy to minimize the energy wasting of amino acids and lipids during hibernation, which is accompanied by a prolonged period of disuse and starvation.

## Introduction

“Use it or lose it” phenomenon is a very well-characterized physiological principle in skeletal muscles, in which an increased muscle contractile activity (e.g., resistance exercise) promotes muscle protein synthesis and hypertrophy, while a decrease in muscle activity level (e.g., prolonged bed rest or joint casting) causes an enhancement of the proteolytic system. This results in progressive muscle loss and subsequent metabolic disorders in many animal species, including human subjects^1,2^. Yet, this physiological principle does not seem to apply to hibernating animals; as if the applicable rule is “no use but no loss”. Hibernating animals can maintain muscle mass and locomotor function despite experiencing prolonged periods of inactivity and malnutrition during the winter denning season^3,4^. To support this idea, previous reports indicated that muscle mass and strength are well preserved during winter survival in hibernating animals. The small hibernating mammals such as the ground squirrel, that repeat multi-day bouts of torpor/hypothermia and brief (about half to one day) interbout arousals cycles during hibernation, exhibit a pronounced decrease in body fat mass but show minimal loss of lean body mass and muscle fiber size during hibernation^5,6^. Also, in the case of bears, larger hibernating mammals with mild hypothermia (30–36 °C) but a pronounced hypometabolic state (~ 25% of basal metabolic rates) that lasts for several months of hibernation, capacity for muscle force generation and total muscle mass was slightly decreased following hibernation, but the degree of muscle weakness is very limited compared to the predicted human response^7–9^. These physiological characteristics of hibernating animals indicate that elucidating the mechanisms of maintaining muscle mass during hibernation is promising for the development of new strategies for preventing muscle loss and the maintenance of physical functions in non-hibernating animals, including humans.

The skeletal muscles represent about 40% of the total body weight and play critical roles in protein storage and energy metabolism in the human body^10^. In the skeletal muscle of hibernating animals, protein turnover during hibernation is potentially decreased, suggesting that this suppressed level of muscle protein metabolism may contribute to the maintenance of muscle mass^11^. A previous study that directly measured muscle protein metabolism in American black bears using a tracer method with radioisotopes showed that, while both protein biosynthetic and degradative pathways were decreased in the skeletal muscles of the bears during the winter hibernation compared to the summer active period, both of them remained in a state of equilibrium and, consequently, muscle mass is maintained^12^. Focusing on bear skeletal muscles, very limited information is currently available on hibernation-associated alterations in the intracellular pathways regulating muscle protein turnover and energy metabolism. Some previous studies have indicated that a gene set involved in protein biosynthesis and ribosome biogenesis is potentially upregulated whereas genes involved in proteolysis and muscle atrophy are suppressed in the skeletal muscles of hibernating American black^13^ or grizzly bears^14^. A proteomic study of skeletal muscles in Swedish brown bears reported that lipid oxidation levels in skeletal muscles were reduced while glycolysis was maintained during hibernation^15^. In our previous study examining alterations in intracellular signaling pathways that regulate protein and energy metabolism in the skeletal muscles of Asiatic black bears, we reported that the mechanistic target of rapamycin (mTOR)-dependent signaling pathway [increased phosphorylation of ribosomal protein S6 kinase B1 (S6K1)] that positively regulates protein synthesis and proteolytic systems, including the ubiquitin–proteasome [increased gene expression of muscle RING finger protein 1 (Murf1)] and autophagy-lysosome systems [increased gene expression of autophagy related 7 (Atg7), beclin 1 (Becn1), and microtubule-associated protein 1 light chain 3 (Map1lc3)] were potentially activated in skeletal muscles following hibernation^8^. These earlier studies, including ours, however, exhibited some potential limitations, which include: (1) lacking detailed analysis of signal transduction systems regulating protein and energy metabolism, (2) a lack of sex control due to the use of wild animals, and (3) the potential acute effects on nutritional status and physical activity during the experimental period have not been eliminated. Therefore, to exclude the potential limitations associated with earlier studies, we examined the alterations in regulatory systems of protein and energy metabolism in the skeletal muscles of bears during the summer active and winter hibernation periods. Another major strength of this study is the comparison of the active and hibernating periods using identical individual bears that were strictly managed in a specialized facility, and not in the wild.

## Results

### No changes in muscle fiber size between active and hibernating periods

**Figure 1:**
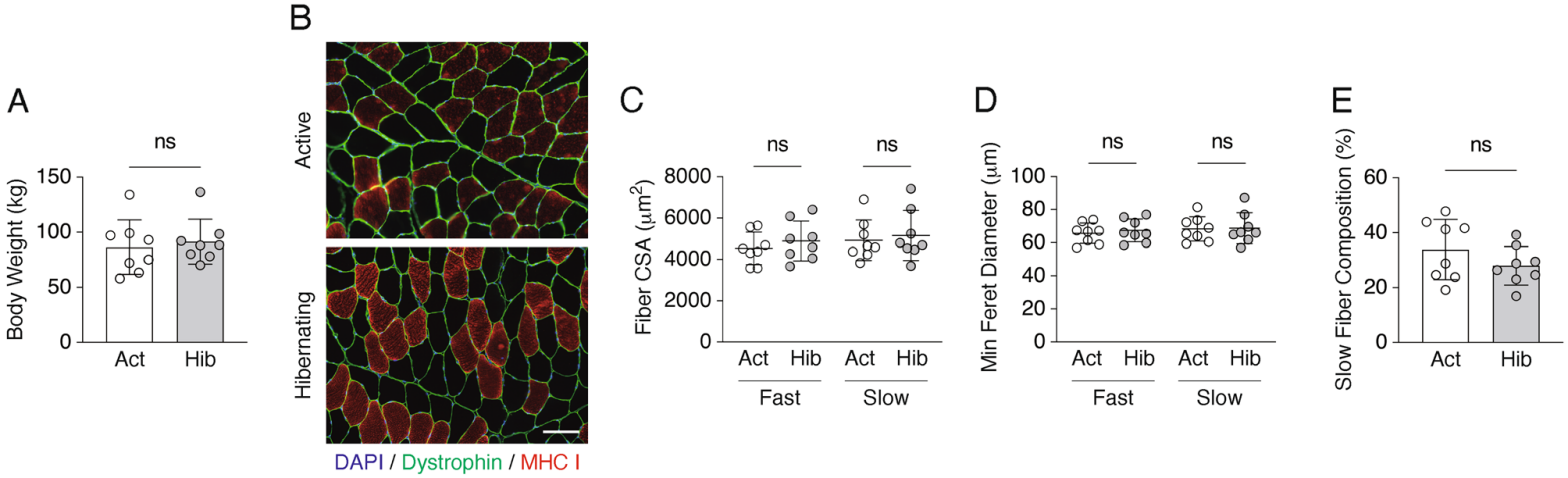
Body weight and muscle fiber cross-sectional areas: active vs. hibernation periods. The body weight, muscle fiber size, and fast-/slow-fiber type composition were not altered in Asiatic black bears during hibernation. During the 2017–2018 season, skeletal muscle samples (vastus lateralis muscle) were collected from each of the same individual bear under anesthesia in mid-July (normal activity period) and in late February (hibernation period). n = 8 in each group. (**A**) Body weight (kg). (**B**) Typical images of cross sections with immunohistochemistry (blue; nuclei localization with 4′,6-diamidino-2-phenylindole, green; dystrophin localization, red; localization of slow-type fiber with anti-myosin heavy chain slow antibody) are shown. The scale bar shows 100 μm. The mean size (**C**) and minimum Feret diameter (**D**) of muscle fibers were obtained for each sample followed by the determination of group data. (**E**) The composition (%) of slow muscle fibers was calculated by counting a total of 400–800 myofibers from different regions in each sample. All results are expressed as mean ± standard deviation (SD).

In this study, we first examined whether body weight and muscle fiber size were altered by comparing summer active versus winter hibernating periods in Asiatic black bears (Fig. 1). Comparing these two points, no change in total body weight was observed. Muscle fiber cross-sectional area (CSA) and minimum Feret diameter were also unchanged between the active and the hibernating periods for both fast- and slow-type fibers. In addition, the composition of muscle fiber types showed no hibernation-associated alterations in the ratio of fast and slow muscle types (Fig. 1 and Supplemental Table 1).

### Alterations in the regulatory systems for muscle protein synthesis during hibernation

**Figure 2:**
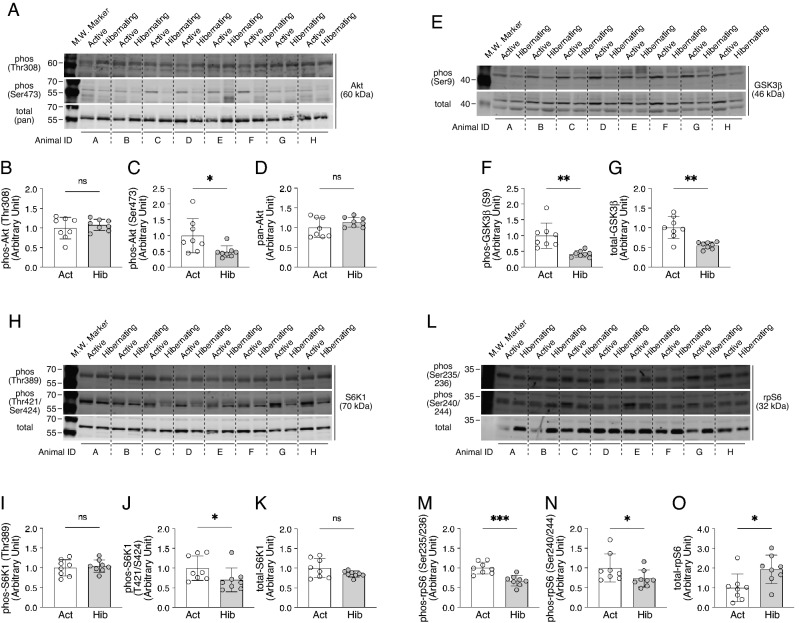
Alterations in the regulatory systems for muscle protein synthesis during hibernation in bears. Typical western blotting images of signaling molecules involved in the regulatory systems for muscle protein synthesis and their quantification results are presented. (**A**–**D**) phosphorylated (Thr308 and Ser473) and total expression of Akt. (**E**–**G**) phosphorylated (Ser9) and total expression of GSK3β. (**H**–**K**) phosphorylated (Thr389 and Thr421/Ser424) and total expression of S6K1. (**L**–**O**) phosphorylated (Ser235/236 and Ser240/244) and total expression of rpS6. n = 8 in each group. All results are expressed as mean ± standard deviation (SD). Significant differences: *p < 0.05, **p < 0.01, ***p < 0.001 between active versus hibernation period.

**Figure 3:**
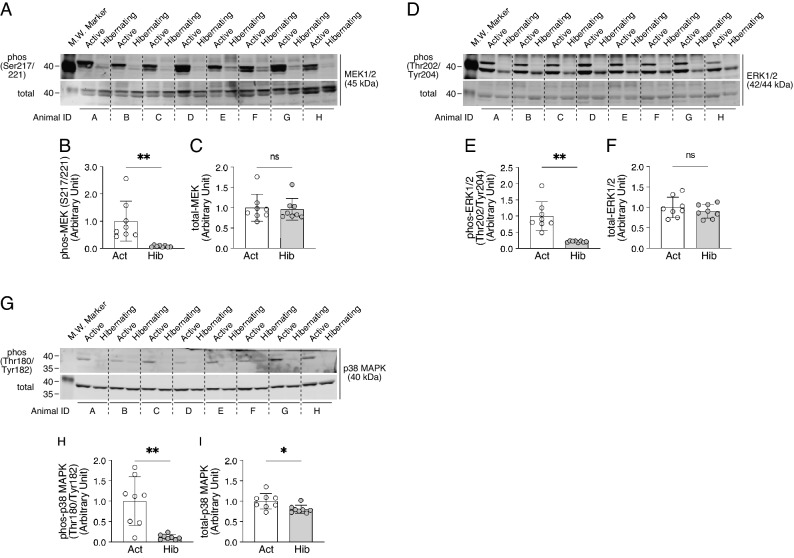
Alterations in the phosphorylation status of MAPK-dependent signaling in bear skeletal muscles during hibernation. Typical western blotting images of signaling molecules involved in MAPK-dependent signaling and their quantification results are presented. (**A**–**C**) phosphorylated (Ser217/221) and total expression of MEK1/2. (**D**–**F**) phosphorylated (Thr202/Tyr204) and total expression of ERK1/2. (**G**–**I**) phosphorylated (Thr180/Tyr182) and total expression of p38 MAPK. n = 8 in each group. All results are expressed as mean ± standard deviation (SD). Significant differences: *p < 0.05, **p < 0.01 between active versus hibernation period.

Subsequently, having found no change in total body weight and muscle fiber size between the active and the hibernating periods in bears, we analyzed how the regulatory system for muscle protein synthesis is altered during hibernation (Fig. 2). As for the Akt-glycogen synthase kinase 3 (GSK3) axis of the protein synthesis system, which plays a pivotal role in regulating translation initiation and elongation, phosphorylation of both Akt (Ser473) and GSK3β (Ser9) were significantly decreased in skeletal muscle during hibernation. Another important regulatory system of protein synthesis, the mTORC1-dependent signaling pathway, showed that the phosphorylation status of S6K1 (Thr421/Ser424) and its downstream effector ribosomal protein S6 (rpS6) (Ser235/236 and Ser240/244), which represents the functional activity of mTORC1 signaling, was significantly decreased in skeletal muscle during hibernation. Conversely, the total protein content of rpS6, a potential indicator of intracellular ribosomal content, was significantly increased during hibernation. Furthermore, the activation status of the mitogen-activated protein kinase kinase (MEK)/extracellular signal-regulated kinase (ERK)-dependent pathway, which is involved in the mTORC1-dependent/independent regulation of muscle protein synthesis, was significantly suppressed with hibernation, as was the reduced phosphorylation status of both signaling molecules (MEK at Ser217/221 and ERK1/2 at Thr202/Tyr204) (Fig. 3A–F). MEK and ERK1/2 phosphorylation are generally considered as an indicator of the mechanical loading of skeletal muscles^16,17^; hence, it can be considered that a reduction in the phosphorylation status of MEK/ERK reflects a decreased muscle contractile activity or a decreased mechanical loading of the skeletal muscles during hibernation. We also examined the phosphorylation of p38, another MAPK family member that is implicated in myogenesis and the differentiation and proliferation of myogenic cells, and found a marked decrease in phosphorylation associated with hibernation in the skeletal muscles of bears (Fig. 3G–I). Overall, these data imply that, although the indicator of ribosomal content (i.e., total rpS6 protein) was increased, the signaling inputs that positively regulate muscle protein synthesis and cell growth were significantly suppressed in the skeletal muscle of bears during hibernation.

### Alteration in the regulatory system for muscle protein catabolism during hibernation

**Figure 4:**
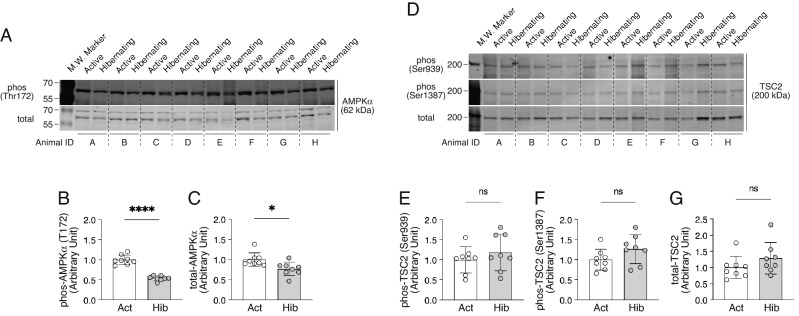
Alterations in the regulatory systems for energy metabolism in bear skeletal muscles during hibernation. Typical western blotting images of signaling molecules involved in the regulatory systems for energy metabolism and their quantification results are presented. (**A**–**C**) phosphorylated (Thr172) and total expression of AMPKα. (**D**–**G**) phosphorylated (Ser939 and Ser1387) and total expression of TSC2. n = 8 in each group. All results are expressed as mean ± standard deviation (SD). Significant differences: *p < 0.05, ****p < 0.0001 between active versus hibernation period.

AMP-activated protein kinase (AMPK), a cellular energy sensor, is activated by increased ADP or AMP concentrations which are associated with ATP hydrolysis and it maintains energy homeostasis by suppressing mitochondrial energy production and promotes the catabolic pathway to generate ATP. As hibernation is considered to be a prolonged period of fasting and energy deprivation, we first expected that AMPK phosphorylation status in skeletal muscles would be elevated during hibernation. However, the reality was the opposite: total expression and phosphorylation of AMPK were significantly suppressed during hibernation in bear skeletal muscles (Fig. 4). This probably indicates that the muscles, during hibernation, are in an energy-saving state, in which ATP hydrolysis is not actively occurring. Since AMPK is known to negatively regulate the mTORC1 signaling pathway through TSC complex subunit 2 (TSC2) or regulatory associated protein of MTOR, complex 1 (Raptor), alterations in TSC2 phosphorylation during hibernation were also examined (phosphorylation of Raptor was also attempted, but could not be detected in bear skeletal muscle samples). We did not observe significant alterations in phosphorylation status and total protein expression of TSC2 in both active and hibernation periods, suggesting that the decreased phosphorylation of AMPK was not causing a compensatory activation of the mTORC1 pathway during hibernation in bear skeletal muscles.

**Figure 5:**
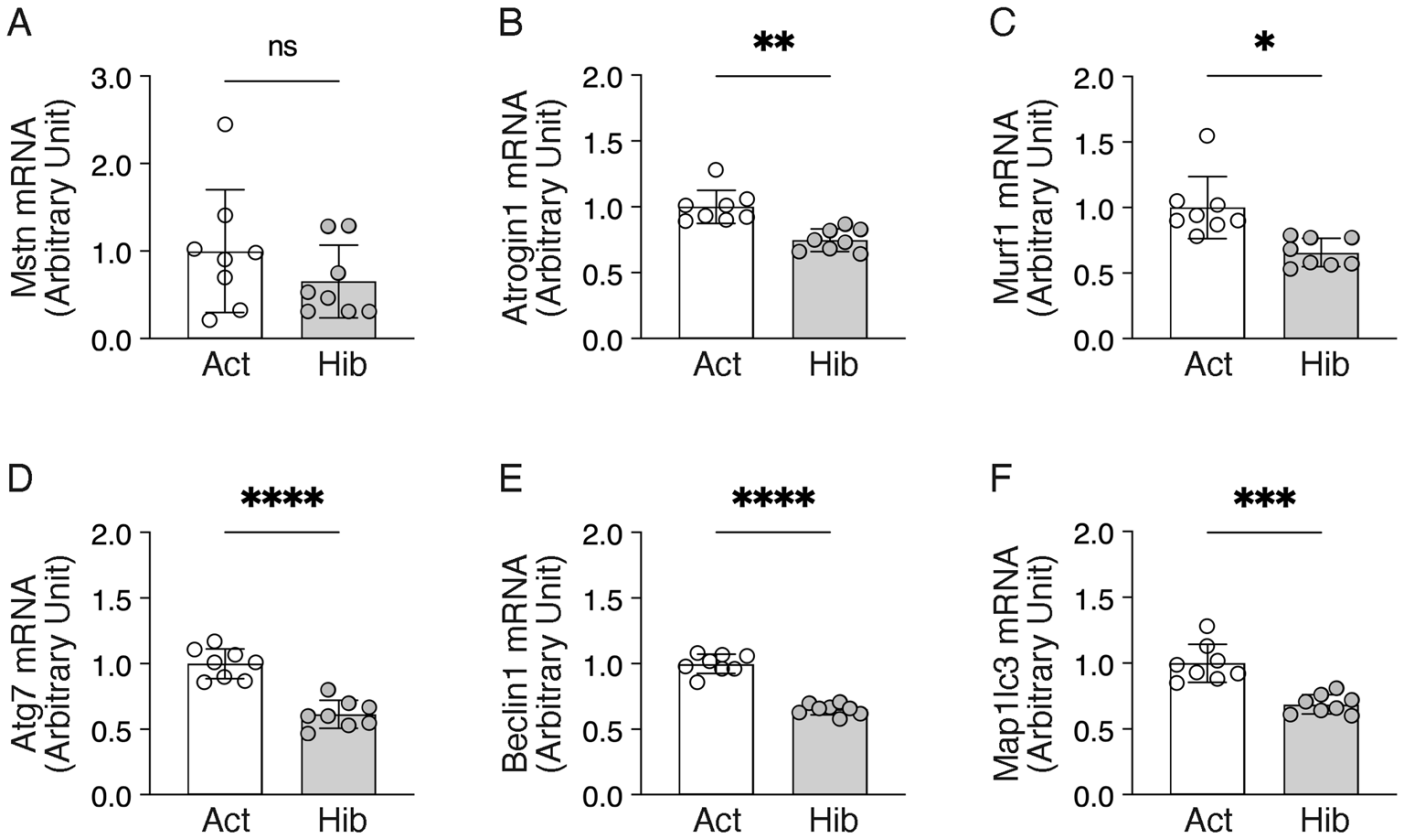
Gene expression of the regulatory systems for muscle protein catabolism during hibernation in bears. Gene expression levels of the myostatin (**A**), ubiquitin–proteasome system (atrogin1 and murf1) (**B**,**C**) and autophagy-lysosome system (atg7, beclin1, and map1lc3) (**D**–**F**) were quantified by real-time PCR. Ribosomal protein L26 was used as an internal control for the 2^–ΔΔCT^ method. n = 8 in each group. Data are expressed as mean ± standard deviation (SD). Significant differences: *p < 0.05, **p < 0.01, ***p < 0.001, ****p < 0.0001 between Active versus Hibernation period.

In addition, the gene expression level of myostatin, a well-known negative regulator of skeletal muscle growth, was determined, and we did not observe any significant alterations during hibernation (Fig. 5A). Further, the expression levels of each gene, which is generally enhanced during skeletal muscle atrophy, in the proteolytic system were determined (Fig. 5B–F). Atrogin1 and Murf1, skeletal muscle-specific E3 ubiquitin ligases, also known as markers of muscle atrophy, showed significantly decreased gene expression levels in skeletal muscles during hibernation. Gene expression levels of autophagy-related factors including Atg7, Becn1, and Map1lc3, were also significantly down-regulated during hibernation. From these results, it is possible to speculate that the ubiquitin–proteasome- and autophagy-lysosome-dependent proteolytic systems are in an inactive state during hibernation in bear skeletal muscles.

### Alterations in the regulatory system for muscle energy metabolism during hibernation

**Figure 6:**
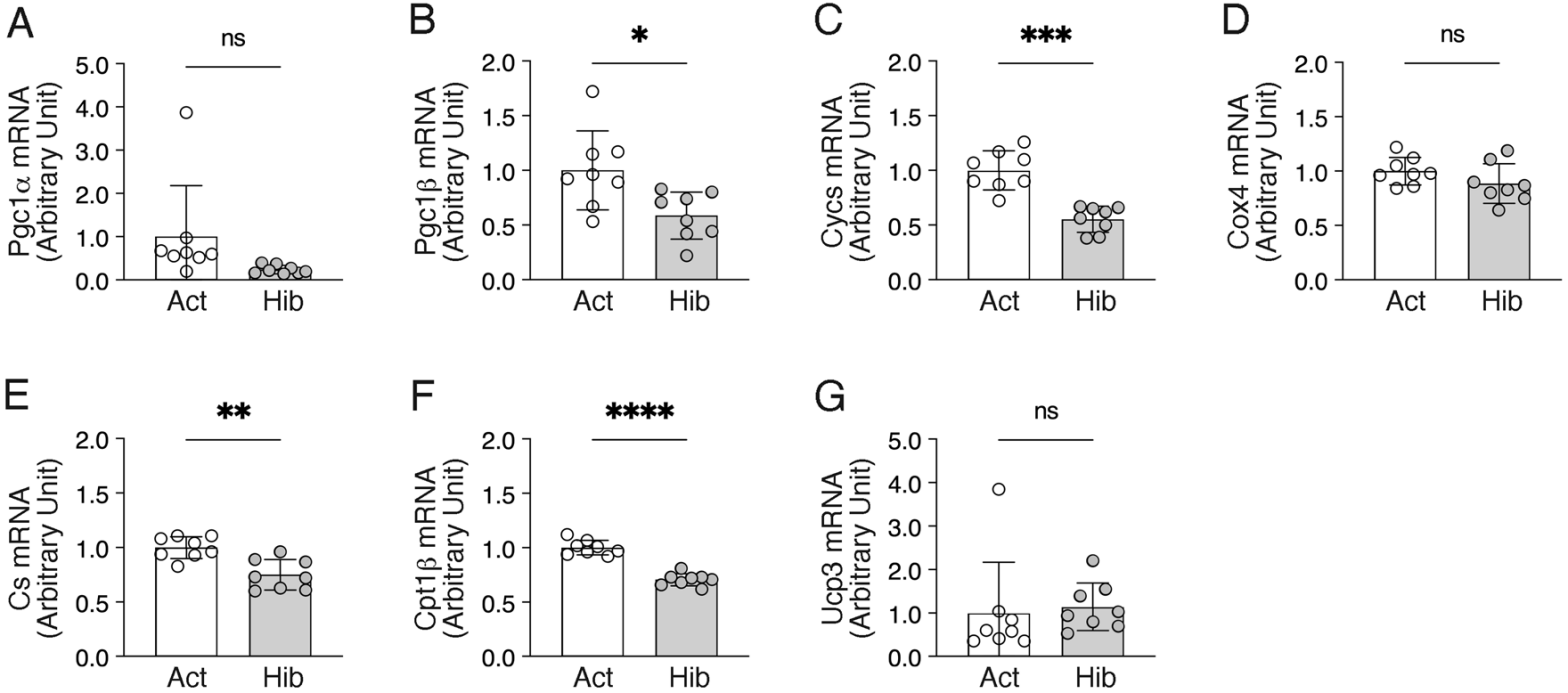
Expression of the mitochondria-related genes in bear skeletal muscles during hibernation*.* Gene expression levels of the peroxisome proliferator-activated receptor gamma coactivator 1 (Pgc1)-alpha (**A**), Pgc1b (**B**), cytochrome c (**C**), cytochrome c oxidase 4 (**D**), citrate synthase (**E**), carnitine palmitoyltransferase 1b (**F**), and uncoupling protein 3 (**G**) were quantified by real-time PCR. Ribosomal protein L26 was used as an internal control for the 2^–ΔΔCT^ method. n = 8 in each group. Data are expressed as mean ± standard deviation (SD). Significant differences: *p < 0.05, **p < 0.01, ***p < 0.001, ****p < 0.0001 between active versus hibernation period.

**Figure 7:**
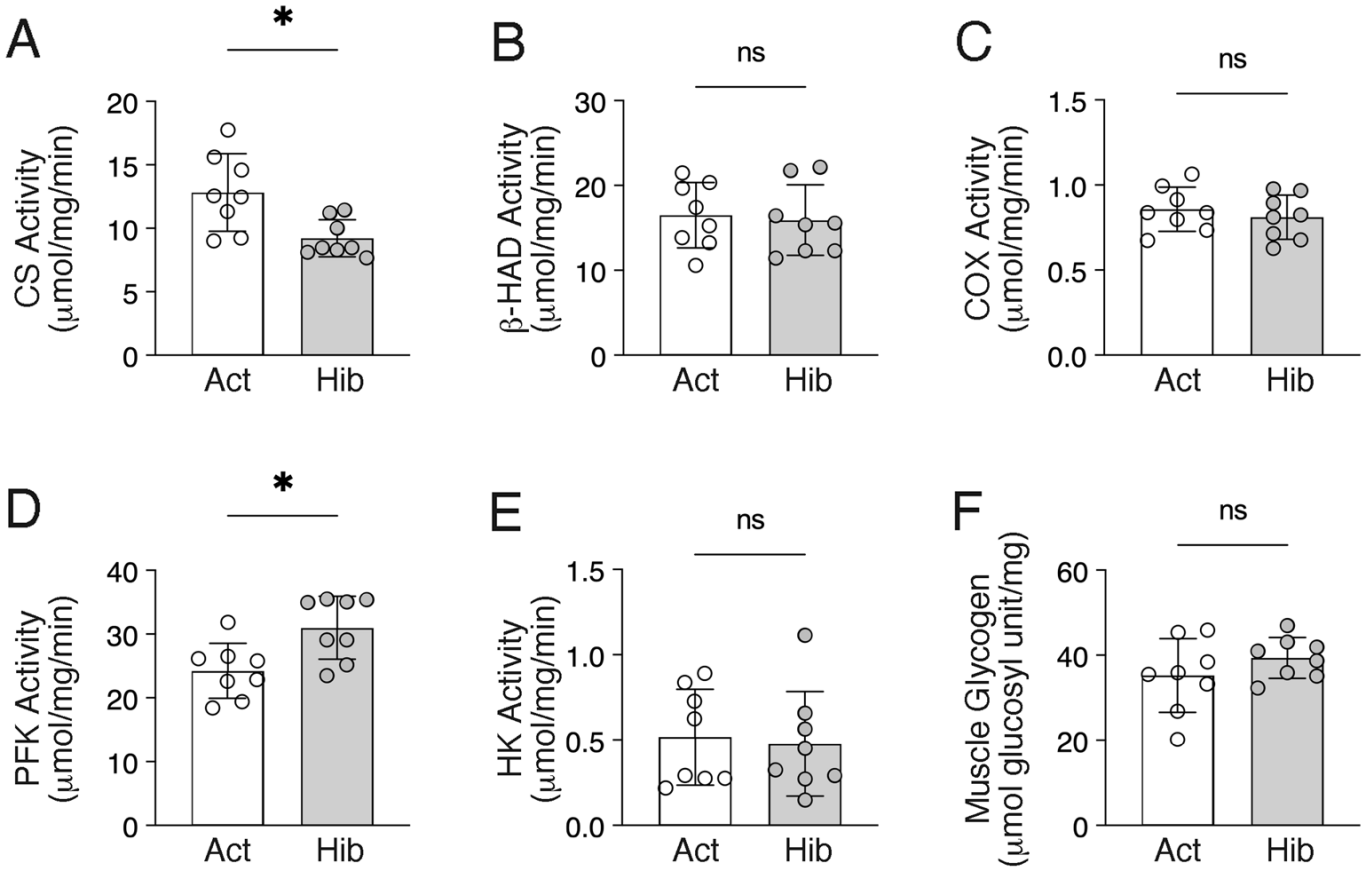
Enzyme activities of oxidative and glycolytic metabolism in bear skeletal muscles during hibernation. Mitochondrial enzyme activities of citrate synthase (**A**), β-HAD (**B**), and cytochrome c oxidase (**C**), along with enzyme activities of glycolysis, including phosphofructokinase (**D**) and hexokinase (**E**) were quantified. (**F**) Muscle glycogen content was determined (mmol glucosyl unit/mg). n = 8 in each group. Data are expressed as mean ± standard deviation (SD). Significant differences: *p < 0.05between active versus hibernation period.

We also evaluated the gene expression levels of regulators associated with mitochondrial biogenesis and oxidative metabolisms (Fig. 6). Although we did not observe significant alterations in the gene expression levels of peroxisome proliferator-activated receptor gamma coactivator 1 alpha (Pgc1α), a master regulator of mitochondrial biogenesis, or uncoupling protein 3, a mitochondrial uncoupling protein, we found that many of the mitochondria-related genes were significantly down-regulated during hibernation in bear skeletal muscles, including the regulator of mitochondrial biogenesis Pgc1β, regulators of electron-transport cytochrome c (Cycs), a rate-limiting enzyme in the TCA cycle, citrate synthase (CS), and fatty acid beta-oxidation regulator carnitine palmitoyltransferase 1b (Cpt1β). Given the suppressed gene expression levels of mitochondria-related factors as described above, then, we examined the alterations in enzyme activities of oxidative metabolism in bear skeletal muscles during hibernation (Fig. 7). As with the gene expression results, the enzymatic activity of CS was significantly decreased in bear skeletal muscles during hibernation. However, no significant alterations were observed in the enzymatic activity of β-hydroxyacyl-CoA dehydrogenase (β-HAD), a key enzyme in β-oxidation, or cytochrome c oxidase (COX), a regulator of the electron-transport system. In addition, based on the observation that mitochondria-mediated oxidative metabolism was decreased in bear skeletal muscles during hibernation, we also determined how glycolytic metabolism is affected. Enzyme activity of phosphofructokinase (PFK), which catalyzes the conversion of fructose-6-phosphate and ATP to fructose-diphosphate and ADP, was significantly increased in bear skeletal muscles during hibernation. Hexokinase (HK), a first-step enzyme in the glycolysis system that phosphorylates glucose, was not changed during hibernation in bear skeletal muscles. Muscle glycogen content was also at the equivalent level during both the active and hibernating periods.

## Discussion

For all living organisms, adaptation to internal and external environmental conditions is an extremely important task for maintaining individual life and the species. For hibernating animals, hibernation is a survival strategy to overcome extreme conditions, such as food deprivation and cold temperatures, during winter. Surprisingly, despite experiencing several months of fasting and physical inactivity, hibernating animals successfully minimize the decline of their physical functions during and following hibernation^3,4^. In our present study, using Asiatic black bears, we observed no significant alterations in muscle fiber size and fiber type composition during active versus hibernating periods, indicating that bear skeletal muscles are very well preserved even after experiencing a prolonged period of inactivity and food deprivation, which is exactly the same trends as previous studies^5,7,8,12,18–20^. In addition, both synthetic (Akt-GSK3-, mTOR-, and MEK/ERK-dependent pathways) and degradative (ubiquitin-proteasomal and lysosome-autophagic pathways) systems of muscle protein metabolisms were significantly down-regulated during hibernation. Furthermore, expression levels of mitochondria-related genes and enzyme activity of oxidative metabolism (CS activity) that regulate the use of lipids, the primary energy source during hibernation in bears, were also significantly decreased. These modifications in bear skeletal muscles are likely adaptations to an energy-saving status, which minimizes the use of energy sources, such as muscle proteins and lipids during hibernation, a period of extremely limited energy availability. The phosphorylation status of AMPK, an energy sensor, was also decreased in hibernating skeletal muscle, suggesting that energy utilization is suppressed by reducing ATP hydrolysis.

Based on the previous study that followed the body weight change of captive Asiatic black bears throughout the year, it is reported that the body weights of the bears were at their highest level just before hibernation in early winter and at their lowest in the post-hibernation state in middle spring^21^. This means that during the summer active period (July) the bears are in an increasing phase of body weight, while during the winter hibernation period (February) they are in a decreasing phase; however, in the comparison between these two phases in this study, there was no change in total body weight. In addition, we have also observed no hibernation-associated alterations in muscle fiber size or muscle fiber composition. Hibernating animals are known to minimize muscle atrophy and maintain muscle mass both before and after the hibernation periods^22^. Although the underlying mechanisms remain unknown, it has long been recognized that the muscle protein contents of hibernating animals are maintained or show minimal loss throughout the hibernation period. Since muscle protein content is determined by the gross balance between protein synthesis and degradation, our finding that both the regulatory systems for protein synthesis and degradation in skeletal muscle were down-regulated during hibernation is a very reasonable explanation for the extraordinary features in hibernating bears that muscle mass is not lost as a result of hibernation. It should be noted, however, that previous studies investigating bear skeletal muscles have shown that the expression levels of genes involved in protein biosynthesis, ribosome biogenesis, or muscle protein anabolic pathways are upregulated during hibernation^13,14,23^. In our previous study, we also observed elevated mTOR signaling, which positively regulates protein synthesis, in bear skeletal muscles at the very end stage of hibernation^8^. These earlier observations may indicate that the regulatory systems involved in protein biosynthesis are potentially upregulated in skeletal muscles during hibernation, which may counteract to prevent an excessive reduction of muscle proteins. Our present results, in which rpS6 phosphorylation was decreased but rpS6 total protein content was increased in hibernating bear skeletal muscles, also support this possible hypothesis.

Currently, there are no consistent observations regarding the expression levels of genes involved in the degradative system in skeletal muscle proteins during hibernation in bears, with reports showing down-regulations during the hibernation periods in grizzly bears^14^, essentially no alterations in American black bears^13^, or an increased level of expression at the end of the hibernation periods in Asiatic black bears^8^. In the present study, which examined seasonal alterations using identical individual bears, we observed hibernation-associated down-regulations in genes involved in the muscle proteolytic pathways, including ubiquitin–proteasome and autophagy-lysosome systems, so it is very likely that the suppression of the proteolytic systems contributes to the maintenance of muscle mass during the hibernation periods. Ex vivo experiments using muscle tissues^24^ or in vitro experiments with muscle cells^25,26^ have demonstrated that the supplementation of hibernating bear serum to the tissue/cell culture medium inhibits the proteolytic systems of the muscles and contributes to an increase in total muscle protein content. In contrast, hibernating bears still maintain skeletal muscle mass even in the denervated state, indicating that the innervation of muscles is not an essential factor for the preservation of muscle mass^18^. Thus, it is very likely that humoral or systemic factors induced during hibernation play a role in the inhibition of muscle protein degradation. The identification of these unknown factors will be important in elucidating the basis of resistance to muscle atrophy in hibernating animals.

Except for food-storing species, such as hamsters, most hibernating animals, including bears, are in a fasting state during hibernation; thus, energy expenditure during hibernation is solely dependent on lipid storage in the body^27–29^. In previous studies focusing on skeletal muscle of small hibernating animals, although there are some conflicting reports, oxidative metabolic enzyme activities and mitochondrial respiration in skeletal muscles decreased during the hibernation periods^20^. Correspondingly, in this study, using bear skeletal muscles, gene expression levels of mitochondrial membrane respiratory chain proteins and oxidative enzyme activity (CS activity) were decreased during hibernation. This suppression in oxidative metabolism may reflect lower systemic energy demands, i.e., hibernation, and contribute to conservations in energy use. Suppression in oxidative metabolism also leads to a reduction in oxidative damage in skeletal muscles. As oxidative damage is a major cause of skeletal muscle atrophy and weakness, this may also contribute to the maintenance of muscle mass during the hibernation periods^30^. Importantly, in this study, among the activities of oxidative enzymes, only CS activity, which is considered an index of mitochondrial content, was significantly decreased, while enzyme activities of β-HAD and COX showed no significant alterations. This could be a possible adaptive mechanism that selectively decreases total mitochondrial contents to minimize total energy expenditure, while maintaining mitochondrial functions, such as β-oxidation and capacity for ATP production through electron transfer systems during hibernation.

While oxidative energy metabolism was suppressed, utilization of the glycolytic system was rather enhanced in hibernating bear skeletal muscles, with a significant increase in PFK activity. This is consistent with the previously-reported proteomic and metabolomic analyses of skeletal muscles from hibernating brown bears^15^, although it differs from previous reports in skeletal muscles of small hibernating animals, which showed that the glycolytic system is down-regulated^31,32^. Importantly, although glucose utilization is potentially enhanced in skeletal muscles during hibernation, the levels of muscle glycogen were identical between the active and hibernating periods. Some earlier reports suggest that hibernating bears activate a Cori cycle that recycles muscle-produced lactate to glucose in the liver^15,33^; hence, there are likely some systems to supplement glycogen into the skeletal muscles.

## Materials and methods

### Antibodies

Phospho-Akt (Thr308, Cat#: 5106), phospho-Akt (Ser473, Cat#: 4060), pan-Akt (Cat#: 4691), phospho-GSK3β (Ser9, Cat#: 5558), GSK3β (Cat#: 9832), phospho-S6K1 (Thr389, Cat#: 9205), phospho-S6K1 (Thr421/Ser424, Cat#: 9204), phospho-S6 ribosomal protein (Ser235/236, Cat#: 4858), phospho-S6 ribosomal protein (Ser240/244, Cat#: 5364), S6 ribosomal protein (Cat#: 2317), phospho-MEK1/2 (Ser217/221, Cat#: 9154), MEK1/2 (Cat#: 4694), phospho-p44/42 MAPK (Erk1/2) (Thr202/Tyr204, Cat#: 4370), p44/42 MAPK (Erk1/2) (Cat#: 4696), phospho-p38 MAPK (Thr180/Tyr182, Cat#: 9216), p38 MAPK (Cat#: 9212), phospho-AMPKα (Thr172, Cat#: 2535), AMPKα (Cat#: 2793), phospho-Tuberin/TSC2 (Ser939, Cat#: 3615), phospho-Tuberin/TSC2 (Ser1387, Cat#: 5584), and Tuberin/TSC2 (Cat#: 4308) were obtained from Cell Signaling Technology (Danvers, MA, USA). S6K1 (Cat#: sc-230) was from Santa Cruz Biotechnology (Santa Cruz, CA, USA). Anti-dystrophin (Cat#: ab15277) was from Abcam (Cambridge, MA, USA). IRDye 800CW Goat anti-Mouse IgG (Cat#: 926-32210) and IRDye 680LT Goat anti-Rabbit IgG (Cat#: 926-68021) were from LI-COR Biosciences (Lincoln, NE, USA). Alexa Fluor 488-conjugated Goat anti- Rabbit IgG (H + L) (Cat#: A11034) and Alexa Fluor 594-conjugated Goat anti-Mouse IgG2b (Cat#: A21145) were from Thermo Fisher Scientific (Rockford, IL, USA). The hybridoma (BA-F8) developed by Schiaffino, S. was obtained from the Developmental Studies Hybridoma Bank, created by the NICHD of the NIH and maintained at The University of Iowa, Department of Biology, Iowa City, IA 52242.

### Animal care and use

All experimental procedures and animal care performed in this study were conducted according to Institutional Guidelines for Animal Care and Use and approved by the Animal Care and Use Committee of the Graduate School of Veterinary Medicine, Hokkaido University (Permit Numbers: Vet17006 and Vet18-0179). The authors ensure this study adheres to ARRIVE guidelines (https://arriveguidelines.org). A total of eight nonpregnant, female Japanese black bears (*Ursus thibetanus japonicus*), between 6 and 23 years of age (17 ± 5.5 years of age, Supplemental Table 2), housed in Ani-Mataginosato Bear Park (Akita Prefecture, Japan, N40° E140.4°) were used for this study. All animal care and handling procedures were followed, as previously described^26,34^. Briefly, animals were fed with dried corn (360 kcal/100 g, approximately 1.5 kg/head) combined with fruits and vegetables as supplements once a day at 16:00 h during the active period (i.e., from late April to late November). Two weeks before or after the fasting period (i.e., late November/early December to early/mid-April) as a transition phase to and from torpor status, the amount of feeding was reduced to one-third (0.5 kg cornmeal/head) compared with the active period. All animals were kept isolated in the indoor dark rooms for denning and had no access to food during the torpor period. Access to drinking water was allowed ad libitum throughout the year.

### Muscle sample collection

During the 2017–2018 season, skeletal muscle samples (vastus lateralis muscle) were collected from each of the same individual bears under anesthesia in mid-July (normal activity period) and in late February (hibernation period). Muscle samples were taken from different sides of the thigh at each time point. Animals were anesthetized with the intramuscular administration of a 3.0 mg/kg zolazepam hydrochloride and tiletamine hydrochloride cocktail, and 40 μg/kg medetomidine hydrochloride using a blow dart shot. Feeding was restricted overnight (approximately 15–16 h) until the anesthesia and sample collection surgery were completed on the following morning. Meloxicam (subcutaneously administered at 0.2 mg/kg for analgesia) and atipamezole hydrochloride (intramuscularly administered at 200 μg/kg as an antagonist to medetomidine hydrochloride) were administered to aid recovery.

### Immunohistochemistry

Muscle samples for immunohistochemical analyses were frozen in liquid nitrogen–cooled isopentane. Cross sections (10 μm) were cut in a cryostat (Microm NX50H, Thermo Fisher Scientific, Rockford, IL, USA) and stored at − 80 °C until analysis. For the immunohistochemical analysis, sections were fixed in 4% paraformaldehyde, permeabilized with 0.1% Triton X-100, and blocked with 1% bovine serum albumin. Mouse anti-myosin heavy chain (MHC slow, alpha- and beta-, BA-F8) and Alexa Fluor 594-conjugated goat anti-mouse IgG2b antibodies were used for detecting the localization of slow-MHC. Rabbit anti-dystrophin and Alexa Fluor 488-conjugated goat anti-rabbit IgG H&L antibodies were used for detecting the localization of dystrophin. Sections were mounted with Vectashield mounting medium with DAPI for microscopic observations. All images were captured using the Keyence BZ-X800 imaging system (Keyence, Osaka, Japan). CSA and minimum Feret diameter of muscle fibers were measured using dystrophin-stained 40X magnification images and imaging software BZ-X800 Analyzer (Keyence, Osaka, Japan). The grid sampling method was used to measure CSAs of muscle fibers, as previously described^35^. Briefly, a cross-section at 40× magnification was taken of a rectangular grid, and all muscle fibers located within the area were sampled for the CSA measurement. Ten to 16 non-overlapped areas were randomly chosen and identically distributed in each whole muscle section such that a total of 400–800 fibers per muscle sample were analyzed. For the determination of the mean fiber CSA in each experimental group, the mean size of the fibers was obtained for each sample before the determination of the group data.

### Protein extraction and western blot analysis

For whole tissue protein extraction, frozen muscle samples were minced and then homogenized in an ice-cold lysis buffer (1% NP-40, 0.5% sodium deoxycholate, 0.1% SDS, 50 mM NaCl, 20 mM Tris–HCl [pH, 7.6], 1 mM PMSF, 5 mM benzamidine, 1 mM EDTA, 5 mM N-ethylmaleimide, 50 mM NaF, 25 mM B-glycerophosphate, 1 mM sodium orthovanadate, and 1× protease inhibitor cocktail [Nacalai Tesque, Kyoto, Japan]). Subsequently, lysed samples were centrifuged at 16,000×*g* for 10 min at 4 °C, and the supernatants were collected for analysis. Protein concentrations were determined using the BCA Protein Assay Kit (Thermo Fisher Scientific, Rockford, IL, USA). Protein samples were separated using a precast polyacrylamide gel system (e-PAGEL; ATTO, Tokyo, Japan) and transferred to PVDF membranes (Immobilon-FL Transfer Membrane; Merck, Darmstadt, Germany). Membranes were then blocked in Odyssey Blocking Buffer (LI-COR Biosciences, Lincoln, NE, USA) and incubated with diluted primary and secondary antibodies. Bound antibody complexes were scanned and quantified using the Odyssey CLx Imaging System operated with Image Studio Version 3.1 software (LI-COR Biosciences, Lincoln, NE, USA). Consistent amount of protein loading was confirmed with using Revert 700 Total Protein Stain Kits (LI-COR Biosciences, Lincoln, NE, USA) (Supplemental Fig. 1). Full-length original images of the blots were shown in Supplemental Fig. 2.

### RNA isolation and real-time PCR

Extraction of total RNA from skeletal muscle samples, cDNA synthesis, and real-time PCR analysis were performed as previously described^8^. Total RNA was extracted using the TRIzol Reagent (Thermo Fisher Scientific, Rockford, IL, USA) according to the manufacturer's instructions. Then, RNA samples were treated with TURBO DNA-free (Thermo Fisher Scientific, Rockford, IL, USA) to remove genomic DNA contamination. Isolated RNA was quantified using spectrophotometry (λ = 260 nm). First-strand cDNA synthesis from total RNA was performed using the PrimeScript RT Reagent Kit. SYBR Premix Ex Taq II and TaKaRa Thermal Cycler Dice Real Time System TP850 (Takara Bio, Shiga, Japan) were used for PCR amplification and quantification of each studied gene. Primer sequences were designed based on partial sequencing of each gene obtained from the Japanese black bear and/or the American black bear^13^ using Primer3 software (Supplemental Table 3). In case the genetic information of black bears cannot be obtained, PCR primers were designed using the genetic information of polar bears (Ursus maritimus, NCBI:txid29073). Expression levels of each of the studied genes were determined by the 2^–ΔΔCT^ method, while referencing ribosomal protein L26 as an internal control.

### Measurement of muscle enzyme activity for energy metabolism

Frozen muscle samples were homogenized in 40 times (vol/wt) of phosphate buffer (100 mM, pH 7.6). Homogenates were subjected to two freeze–thaw cycles to facilitate the release of mitochondrial proteins. After centrifuging at 1000×*g* for 10 min at 4 °C, the supernatant was used for the following enzyme activity assay. HK: HK activity was determined, as previously described, with slight modifications^36^. After the aliquots were mixed with the reaction mixture (50 mM triethanolamine, 5 mM EDTA, 10 mM MgCl_2_, 0.35 mM NADH, 2.8 mM ATP, 2.8 mM glucose, and 2.5 U glucose-6-phosphatase; pH 7.6), the changes in absorbance were measured at 340 nm. PFK: PFK activity was determined, as previously described, with slight modifications^36^. After the aliquots were mixed with the reaction mixture (50 mM triethanolamine, 5 mM EDTA, 10 mM MgCl_2_, 0.3 mM NADH, 2.8 mM ATP, 2.8 mM F-6-P, 2.5 U GPDH-TPI, and 1.0 U aldolase; pH 7.6). The changes in absorbance were measured at 340 nm. CS: CS activity was determined, as previously described, with slight modifications^37^. After the aliquots were mixed with the reaction mixture (100 mM Tris, 100 μM DTNB, 300 μM acetyl-CoA, and 50 μM oxaloacetate; pH 8.3), the changes in absorbance were measured at 412 nm. COX: COX activity was determined, as previously described, with slight modifications^38^. After the aliquots were mixed with the reaction mixture (10 mM phosphate and 50 μM cytochrome c reduced with sodium hydrosulfite; pH 7.0), the changes in absorbance were measured at 550 nm. β-HAD: β-HAD activity was determined, as previously described, with slight modifications^39^. After the aliquots were mixed with the reaction mixture (1 M Tris, 5 mM EDTA, 450 µM NADH, and 100 µM acetoacetyl-CoA; pH 7.0), the changes in absorbance were measured at 340 nm.

### Muscle glycogen quantification

Muscle glycogen content was determined, as previously described^40^. Briefly, the muscle samples were heated at 100 °C in 30% KOH solution saturated with Na_2_SO_4_, mixed with 99.5% ethanol, and then centrifuged at 10,000×*g* for 10 min at 4 °C. The pellets were hydrolyzed to glucose in 1 M HCl at 100 °C for 2 h and neutralized with 1 M NaOH. Glycogen content was determined using a glucose CII kit (Fujifilm Wako, Osaka, Japan).

### Statistical analysis

All results are reported as means ± standard deviation. Statistical differences between the summer active period (active) and the winter hibernation period (Hibernation) were determined using a Student’s t-test. For all comparisons, the level of statistical significance was set at p < 0.05.

## Supplementary Information

Supplementary Figure 1.

Supplementary Figure 2.

Supplementary Table 1.

Supplementary Table 2.

Supplementary Table 3.

Supplementary Legends.

## Data Availability

The all datasets used and/or analyzed in the current study are available from the corresponding author on reasonable request.
