# Supplementary figures and images for: Regulation of protein and oxidative energy metabolism are down-regulated in the skeletal muscles of Asiatic black bears during hibernation
Source: Sci Rep. 2022 Nov 16;12:19723. doi: 10.1038/s41598-022-24251-0 (PMC9668988; doi:10.1038/s41598-022-24251-0)

Supplemental Figure 1

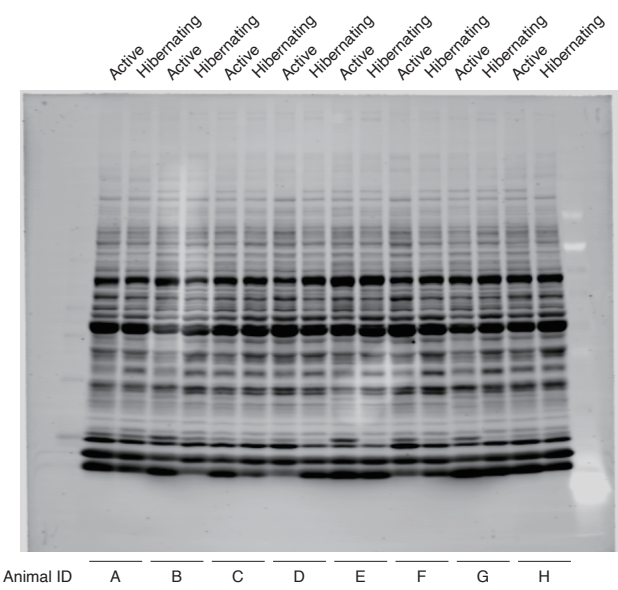

Supplement: Supplementary file 1 — Supplementary Figure 1. [file 41598_2022_24251_MOESM1_ESM.pdf]
