# Supplementary material for: Regulation of protein and oxidative energy metabolism are down-regulated in the skeletal muscles of Asiatic black bears during hibernation
Source: Sci Rep. 2022 Nov 16;12:19723. doi: 10.1038/s41598-022-24251-0 (PMC9668988; doi:10.1038/s41598-022-24251-0)

Supplemental Figure 2

raw_images for Figure 2A

phos-Akt (Thr308)

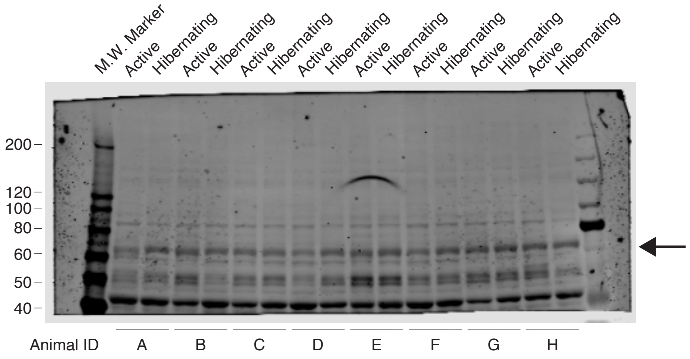

phos-Akt (Ser473)

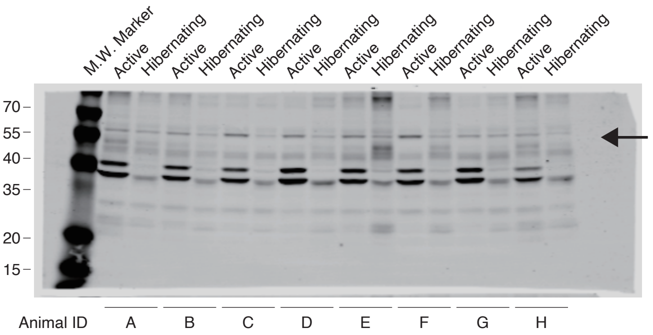

pan-Akt

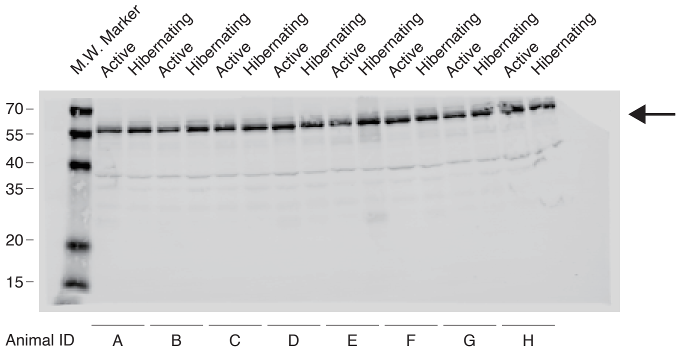

raw_images for Figure 2E

phos-GSK3β (Ser9)

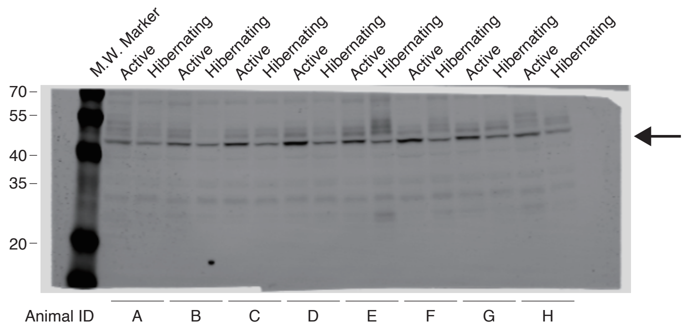

total-GSK3β

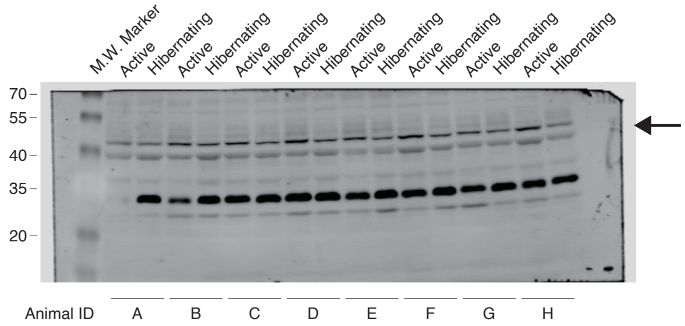

raw_images for Figure 2H

phos-S6K1 (Thr389)

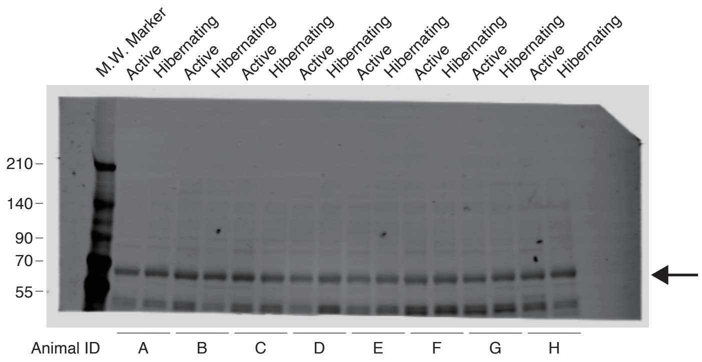

phos-S6K1 (Thr421/Ser424)

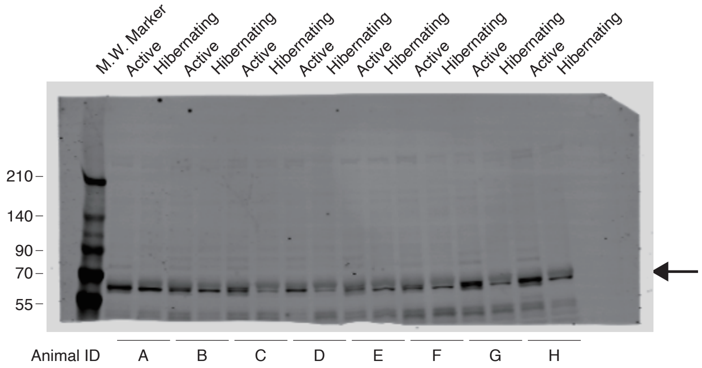

total-S6K1

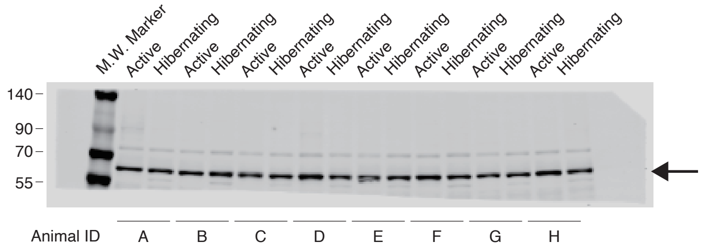

raw_images for Figure 2L

phos-rpS6 (Ser235/236)

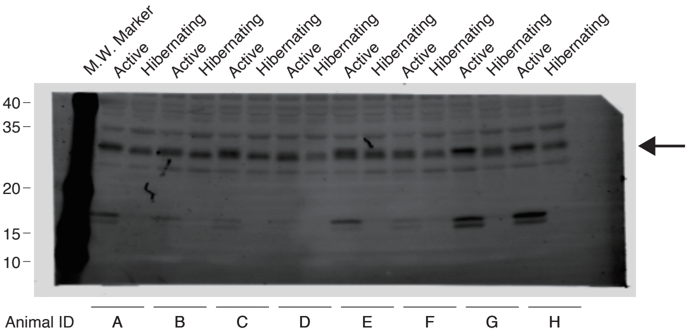

phos-rpS6 (Ser240/244)

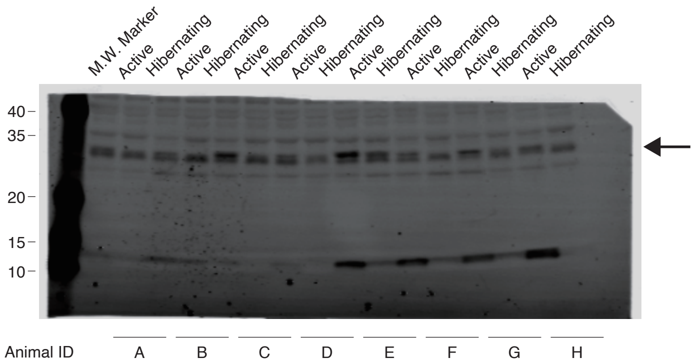

total-rpS6

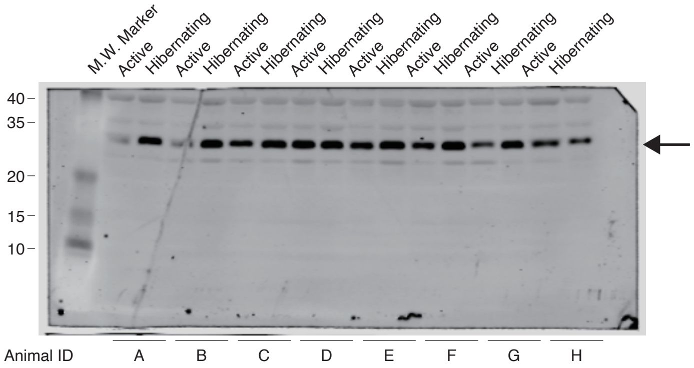

raw_images for Figure 3A

phos-MEK1/2 (Ser217/221)

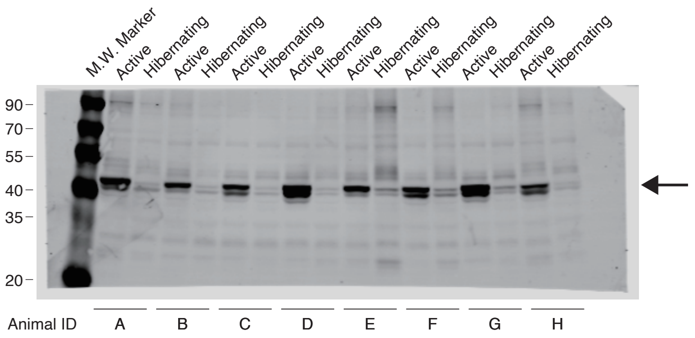

total-MEK1/2

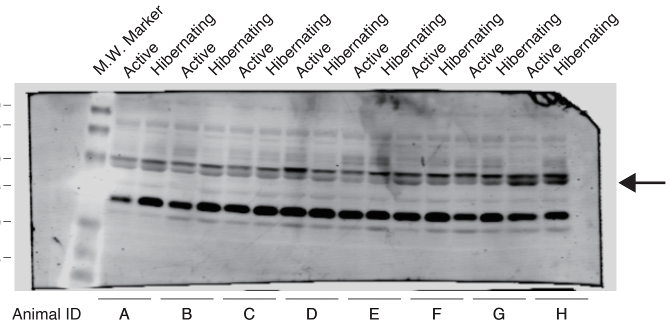

raw_images for Figure 3D

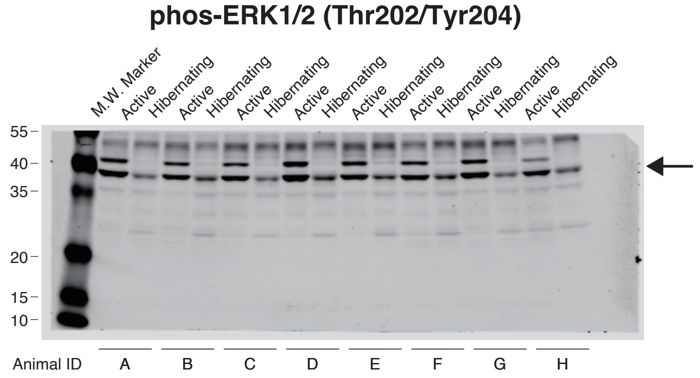

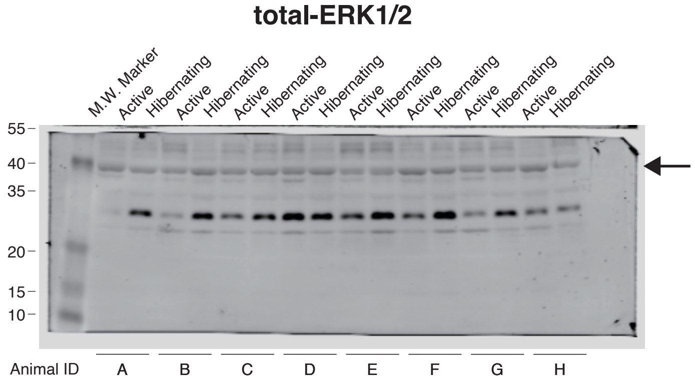

raw_images for Figure 3G

phos-p38 MAPK (Thr180/Tyr182)

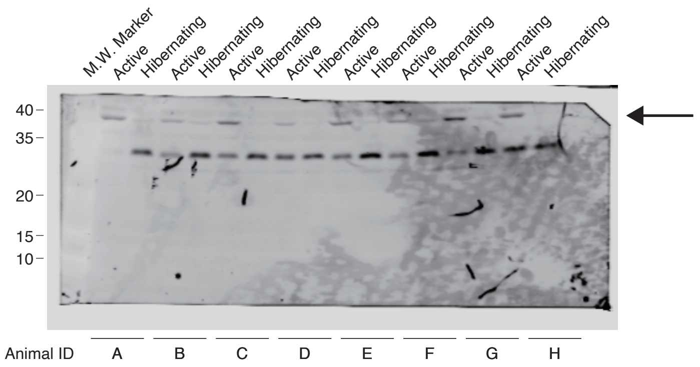

total-p38 MAPK

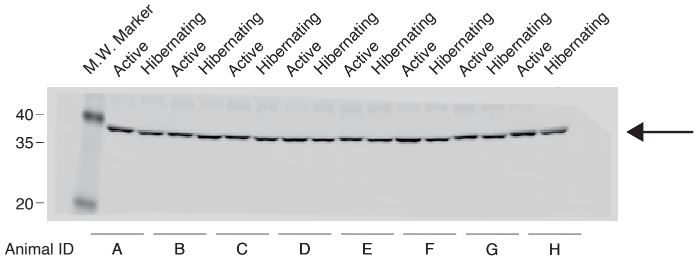

raw_images for Figure 4A

phos-AMPKα (Thr172)

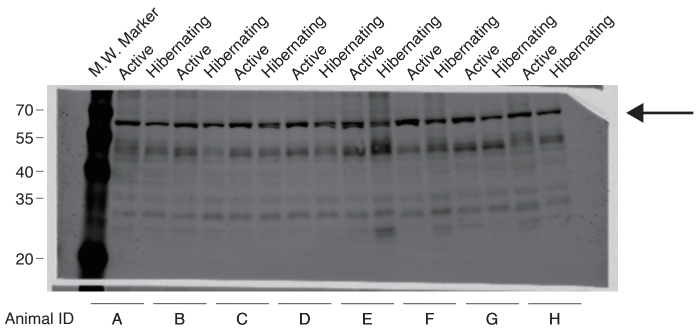

total-AMPKα

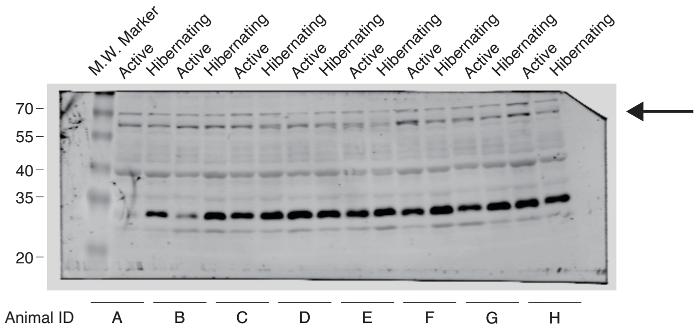

raw_images for Figure 4D

phos-TSC2 (Ser939)

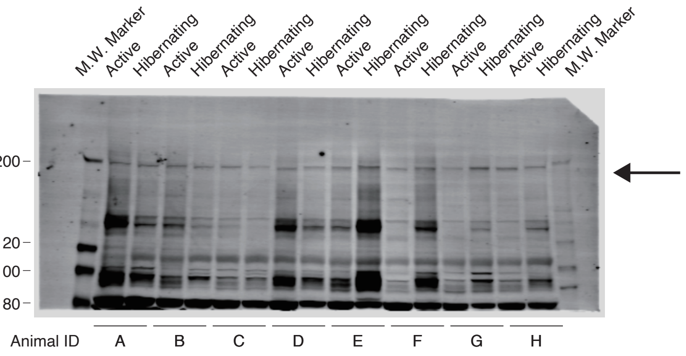

phos-TSC2 (Ser1387)

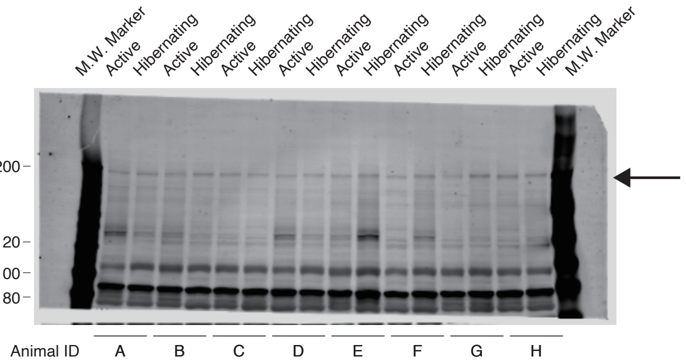

total-TSC2

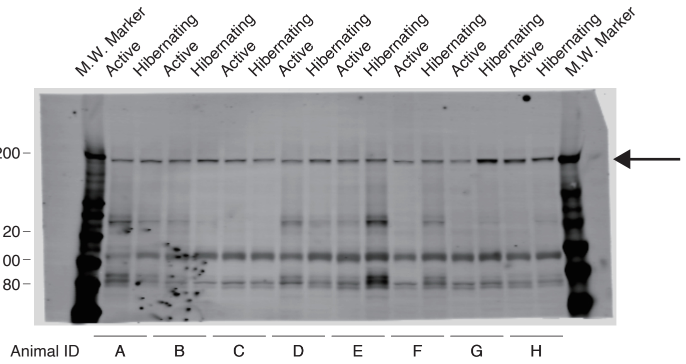

Supplement: Supplementary file 2 — Supplementary Figure 2. [file 41598_2022_24251_MOESM2_ESM.pdf]
