# Supplementary material for: Regulation of protein and oxidative energy metabolism are down-regulated in the skeletal muscles of Asiatic black bears during hibernation
Source: Sci Rep. 2022 Nov 16;12:19723. doi: 10.1038/s41598-022-24251-0 (PMC9668988; doi:10.1038/s41598-022-24251-0)
Supplement: Supplementary file 3 — Supplementary Table 1. [file 41598_2022_24251_MOESM3_ESM.docx]

**Supplemental Table 1. Muscle fiber size and fiber type composition for each individual bear**

| Animal ID | Fast Fiber CSA (μm^2^) | | Slow Fiber CSA (μm^2^) | | Min Feret Dia Fast (μm) | | Min Feret Dia Slow (μm) | | Sow Fiber Comp. (%) | |
| --- | --- | --- | --- | --- | --- | --- | --- | --- | --- | --- |
|  | Act | Hib | Act | Hib | Act | Hib | Act | Hib | Act | Hib |
| A | 5647.0 | 4070.0 | 5691.57 | 4805.87 | 73.87 | 59.96 | 73.70 | 64.97 | 43.93 | 29.53 |
| B | 4315.7 | 6403.6 | 5209.05 | 6450.83 | 67.39 | 77.03 | 72.37 | 77.05 | 41.70 | 35.48 |
| C | 4708.3 | 6097.0 | 6904.86 | 7424.64 | 66.77 | 75.47 | 81.34 | 87.17 | 23.91 | 23.12 |
| D | 4388.7 | 5096.2 | 4240.56 | 4545.29 | 61.55 | 72.12 | 59.38 | 66.36 | 19.24 | 28.92 |
| E | 3582.1 | 4269.3 | 3830.22 | 4694.05 | 58.97 | 65.44 | 60.60 | 69.64 | 28.83 | 39.21 |
| F | 4526.0 | 4597.8 | 4662.13 | 4479.37 | 67.17 | 64.85 | 69.02 | 61.95 | 47.85 | 24.69 |
| G | 3575.6 | 5004.1 | 4593.29 | 5252.32 | 56.98 | 66.05 | 65.54 | 66.12 | 41.25 | 26.42 |
| H | 5576.7 | 3671.7 | 4391.08 | 3689.65 | 73.03 | 58.55 | 64.16 | 56.92 | 24.67 | 16.98 |
