# Supplementary material for: Regulation of protein and oxidative energy metabolism are down-regulated in the skeletal muscles of Asiatic black bears during hibernation
Source: Sci Rep. 2022 Nov 16;12:19723. doi: 10.1038/s41598-022-24251-0 (PMC9668988; doi:10.1038/s41598-022-24251-0)
Supplement: Supplementary file 4 — Supplementary Table 2. [file 41598_2022_24251_MOESM4_ESM.docx]

**Supplemental Table 2. Physical characteristics data for each individual bear**

| Animal ID | Sample Collection Season | Age | Body Weight (kg) | |
| --- | --- | --- | --- | --- |
|  |  |  | Active | Hibernating |
| A | 2017 Feb / 2017 July | 23 | 97.0 | 92.0 |
| B | 2017 Feb / 2017 July | 22 | 58.0 | 70.0 |
| C | 2017 Feb / 2017 July | 22 | 134.0 | 136.5 |
| D | 2017 Feb / 2017 July | 20 | 75.0 | 80.0 |
| E | 2017 July / 2018 Feb | 17 | 63.5 | 88.0 |
| F | 2018 Feb / 2018 July | 14 | 99.0 | 88.5 |
| G | 2018 Feb / 2018 July | 13 | 93.0 | 98.5 |
| H | 2018 Feb / 2018 July | 6 | 72.0 | 78.0 |
