# Supplementary material for: Regulation of protein and oxidative energy metabolism are down-regulated in the skeletal muscles of Asiatic black bears during hibernation
Source: Sci Rep. 2022 Nov 16;12:19723. doi: 10.1038/s41598-022-24251-0 (PMC9668988; doi:10.1038/s41598-022-24251-0)
Supplement: Supplementary file 5 — Supplementary Table 3. [file 41598_2022_24251_MOESM5_ESM.docx]

**Supplemental Table 3. Primer Sequences**

| Gene ID | Gene  symbol | description | Forward | Reverse |
| --- | --- | --- | --- | --- |
| 123798029 | MSTN | myostatin | tgcatgtacttggaggcaaa | agacgaagtttactgaggatttgaa |
| 123797728 | FBXO32 | F-box protein 32  (atrogin1) | tggacaaaattgttcagaaggtc | tcaggtcctggaggaggtc |
| 123782318 | TRIM63 | tripartite motif containing 63  (murf1) | tccagaggcagtaagggaag | tcatcccctcagcgatctt |
| 123801971 | ATG7 | autophagy related 7 | cagtgcttttgacatgagtgc | tggtgttaaagagagttccaacag |
| 123781873 | BECN1 | beclin 1 | ggtgaaaccaggagagactca | cgagtttcgagaaatggctct |
| 123776674 | MAP1LC3B | microtubule associated protein 1 light chain 3 beta | caaaaccaaattccttgtacctg | gctgtaagcgcctcctaatg |
| 123775818 | PPARGC1A | PPARG coactivator 1 alpha | gaactgcaggcctaactcca | gcaagaggacttcagcttcg |
| 123803693 | PPARGC1B | PPARG coactivator 1 beta | gactttcccgagcttgacc | gcaggtggctgagtcaaagt |
| 123789159 | LOC123789159 | cytochrome c | cttacacggatgccaacaag | tgggattctccaaatactcca |
| 123798702 | LOC123798702 | cytochrome c oxidase subunit 4 | atcaccctgacggatgagtg | gcccttcatatccagaatgc |
| 123787250 | CS | citrate synthase | ggcccaatgtagatgctcac | ttcatctctgtcatgccgtagt |
| 123803781 | CPT1B | carnitine palmitoyltransferase 1B | gatgtggcccctacaggac | gaagcagtttcagggtttgg |
| 123804242 | UCP3 | uncoupling protein 3 | ggagccctaaggaaccctaa | gctctgcagtcctgagcttta |
| 123792100 | RPL26 | ribosomal protein L26 | gagcaagaaccgtaaacgaca | tttggaaagaggggaagaca |
