# Supplementary material for: Regulation of protein and oxidative energy metabolism are down-regulated in the skeletal muscles of Asiatic black bears during hibernation
Source: Sci Rep. 2022 Nov 16;12:19723. doi: 10.1038/s41598-022-24251-0 (PMC9668988; doi:10.1038/s41598-022-24251-0)
Supplement: Supplementary file 6 — Supplementary Legends. [file 41598_2022_24251_MOESM6_ESM.docx]

Supplemental Figure 1. Total protein staining of the blot. Consistent amount of protein loading was confirmed with using Revert 700 Total Protein Stain Kits.

Supplemental Figure 2. Full-length original blots. Original images of the blots used in Figures 2 to 4 were shown.
